# Dietary carbohydrate and the risk of type 2 diabetes: an updated systematic review and dose–response meta-analysis of prospective cohort studies

**DOI:** 10.1038/s41598-022-06212-9

**Published:** 2022-02-15

**Authors:** Fatemeh Hosseini, Ahmad Jayedi, Tauseef Ahmad Khan, Sakineh Shab-Bidar

**Affiliations:** 1 Department of Clinical Nutrition, School of Nutritional Sciences and Dietetics, Tehran University of Medical Sciences (TUMS), Tehran, Iran; 2 Social Determinant of Health Research Center, Semnan University of Medical Sciences, Semnan, Iran; 3 Department of Nutritional Sciences, Faculty of Medicine, University of Toronto, Toronto, ON Canada; 4 Toronto 3D Knowledge Synthesis and Clinical Trials Unit, Clinical Nutrition and Risk Factor Modification Centre, St. Michael’s Hospital, Toronto, ON Canada; 5 Department of Community Nutrition, School of Nutritional Sciences and Dietetics, Tehran University of Medical Sciences (TUMS), No 44, Hojjat-dost Alley, Naderi St., Keshavarz Blvd, P. O. Box 14155/6117, Tehran, Iran

**Keywords:** Diseases, Medical research

## Abstract

We did this study to clarify the association between carbohydrate intake and the risk of type 2 diabetes (T2D) and potential effect modification by geographical location. PubMed, Scopus and Web of Science were searched to find prospective cohort studies of dietary carbohydrate intake and T2D risk. A random-effects dose–response meta-analysis was performed to calculate the summary hazard ratios (HRs) and 95%CIs. The quality of cohort studies and the certainty of evidence was rated using the Newcastle–Ottawa Scale and GRADE tool, respectively. Eighteen prospective cohort studies with 29,229 cases among 607,882 participants were included. Thirteen studies were rated to have high quality, and five as moderate quality. The HR for the highest compared with the lowest category of carbohydrate intake was 1.02 (95%CI: 0.91, 1.15; I^2^ = 67%, GRADE = low certainty). The HRs were 0.93 (95%CI: 0.82, 1.05; I^2^ = 58%, n = 7) and 1.26 (95%CI: 1.11, 1.44; I^2^ = 6%, n = 6) in Western and Asian countries, respectively. Dose–response analysis indicated a J shaped association, with the lowest risk at 50% carbohydrate intake (HR_50%_: 0.95, 95%CI: 0.90, 0.99) and with risk increasing significantly at 70% carbohydrate intake (HR_70%_: 1.18, 95%CI: 1.03, 1.35). There was no association between low carbohydrate diet score and the risk of T2D (HR: 1.14, 95%CI: 0.89, 1.47; I^2^ = 90%, n = 5). Carbohydrate intake within the recommended 45–65% of calorie intake was not associated with an increased risk of T2D. Carbohydrate intake more than 70% calorie intake might be associated with a higher risk.

## Introduction

Type 2 diabetes (T2D) is a serious non-communicable chronic disease described by impaired insulin action or secretion or impaired response of body cells to insulin, followed by the endocrine pancreas' incapability to compensate for this weakened response^1,2^. It is estimated that at least 514 million people are affected by T2D all over the world^1^. The Middle East and North Africa is a region with the highest prevalence of T2D across the globe^2,3^. T2D is a chronic progressive disease, and therefore, lifestyle modifications such as dietary interventions, physical activity, and weight reduction are the core part of first-line interventions for the prevention of T2D^4–6^.

Dietary factors might have a role in the development of T2D^7^. Of note, dietary carbohydrates have received specific attention because of their effect on blood glucose level^8^. Dietary carbohydrates are the major dietary energy source^9,10^ and have the greatest impact on postprandial blood glucose levels^11^. Studies have shown that glycemic properties of the diet including glycemic index and load might be associated with the risk of developing T2D and other chronic diseases^11^.

With regard to T2D, three meta-analyses of cohort studies have been undertaken of the association between dietary carbohydrate and the risk of T2D, but the results have been inconsistent^11–13^. However, almost all studies included in the published meta-analyses have been conducted in Western countries, where the intake of carbohydrates was lower than that of Asian countries^14,15^. A recent publication from the PURE study in 21 countries across the world indicated that higher rice consumption was associated with a greater risk of developing T2D, with the strongest association in South Asia and a modest, nonsignificant association in other regions^16^.

A number of population-based prospective cohort studies in Asian countries have recently been published that reported significant positive associations between dietary carbohydrates and the risk of T2D^17–19^. In the current study, we therefore aimed to update the evidence from prospective cohort studies of the association between dietary carbohydrate intake and low-carbohydrate diet score (LCDS) with the risk of T2D in the general population. Our secondary outcome was to assess this association separately in Asian and Western countries.

## Martials and methods

The Meta-Analysis of Observational Studies in Epidemiology (MOOSE) guidelines have been used for reporting this meta-analysis^20^. The protocol of the study was registered at Open Science Framework (https://osf.io/tvam2; registered form: osf.io/4vu5s; registration https://doi.org/10.17605/OSF.IO/TVAM2).

### Search strategy

We performed a comprehensive systematic search on all literature issued earlier than April 2021 in online databases including PubMed/Medline, Scopus and ISI Web of Science. We did not exert any limitation in term of language or time of publication. We used search terms relevant to type 2 diabetes, carbohydrate, and study design to find potential eligible cohort studies (Supplementary Table 1). Reference lists of retrieved articles and relevant reviews were also manually searched. Unpublished data was not included.

### Inclusion and exclusion criteria

Relevant articles with all of the following inclusion criteria were included: (1) published prospective cohort studies conducted in the general population; (2) reported carbohydrate consumption (as either g/d or percentage energy) and LCDS as exposure; (3) considered T2D incidence as the outcomes of interest; (4) provided estimates of the effect size in the form of relative risk, hazard ratio (HR) or rate ratio with corresponding 95% confidence intervals (CIs) for ≥ 2 quantitative categories of carbohydrate consumption or LCDS; and (5) provided the numbers of cases and non-cases or person-years in each category of dietary carbohydrate or LCDS. Studies that reported continuous estimation from the associations were also included. For duplicate publications form the same cohort, the one with the greater number of cases was included in our meta-analysis. We excluded letters, comments, reviews and meta-analyses, and ecologic studies. We also did not include studies that were performed on children or adolescences or those that were conducted among patients with type one diabetes. All outcomes were classified based on the World Health Organization’s international classification of disease criteria.

### Data extraction

Data extraction process was executed by two reviewers in duplicate (FH and AJ), and any divergences were resolved by consultation the principal investigator (SS-B). We extracted the following information from the publications identified: name of the first author, publication year, country, age, sex, study participants, number of cases, duration of follow-up, method of assessment of carbohydrate consumption and LCDS, the fully-adjusted estimates and their 95%CI and list of potential confounders entered into the multivariable statistical model. Gender-specific estimates were combined a by fixed-effects model to include each cohort once in the main analysis. We used web plot digitizer (http://plotdigitizer.sourceforge.net/) to extract numerical estimates from graphs.

### Data synthesis and analysis

We considered the HR and its 95%CI as the effect size for the present study. Relative risks were considered equal to HR^21^. We first performed a pairwise meta-analysis by combining the reported effect sizes for the highest compared with the lowest category of dietary carbohydrate or LCDS in each study. Study-specific results were combined with a random-effects model^22^. The Cochran Q^23^ and I^2^ statistic^24^ were used to test for presence of heterogeneity.

Subgroup analyses of dietary carbohydrates were performed based on sex, geographic location, number of cases, duration of follow-up and adjustments for main confounders including body mass index (BMI), smoking status, alcohol drinking, and energy and fiber intakes. *P* value for subgroup difference was generated using meta-regression analysis. Subgroup analyses of LCDS were performed based on sex, study location, and duration of follow-up. Publication bias was assessed by visual inspection of funnel plot^23^ and Egger’s^25^ and Begg’s^26^ tests, when at least 10 studies were available. To determine whether the pooled effect size was influenced heavily by a single cohort, sensitivity analysis was done by step-by-step omission of each cohort at a time.

We used the method introduced by Greenland^27^ and Orsini^28^ for dose–response meta-analysis. We calculated the HRs for a 10% increment in carbohydrate intake or a 10-point increment in LCDS in each study. Study-specific HRs were combined by a random-effects model. For this purpose, each cohort study must report the number of cases and person-years and median or range of dietary carbohydrate or LCDS across categories of exposures. For studies that reported dietary carbohydrate as g/d, we converted g/d to percentage calorie from carbohydrate by using the average daily energy intake of the study participants. For studies that reported the results per unit increment in dietary carbohydrate (i.e., per 200 g/d increment), we first converted g/d to percentage energy from carbohydrate and then translated it to a 10% increment in energy intake from carbohydrate. For studies that used different units (for example, 5% increase in carbohydrate intake), we calculated the logarithm of the HR and its 95%CI, multiplied by the corresponding unit, and then exponentiated the results. For studies that reported carbohydrate intake as a range in each category, we used the midpoint of lower bounds as a proxy of the median. The widths of the open-ended categories were considered equal to the closest categories.

Finally, we performed a one-stage weighted mixed-effects meta-analysis to model dose–response associations^29^. This method estimates the study-specific slope lines and combines them to obtain an overall average slope in a single stage. We included all studies in the main analysis. However, due to substantial difference in carbohydrate consumption in Asian and Western countries, we performed separate nonlinear dose–response analyses in Asian and Western countries. Statistical analyses were conducted using STATA software, version 15.0. *P* < 0.05 was considered statistically significant.

### Quality assessments and grading the evidence

The quality of the original studies included in the present meta-analysis was evaluated using a 9-point Newcastle–Ottawa Scale by two independent investigators (FH and AJ)^30^. Accordingly, studies with 1–3, 4–6, and 7–9 points were rated as poor, fair, and high quality, respectively. The certainty in the estimates was rated by the Grading of Recommendations Assessment*,* Development, and Evaluation (GRADE) approach. GRADE tool is a metric to assess the certainty of the evidence^31^. This tool grades observational studies as low with downgrades for study limitations, inconsistency, indirectness, imprecision, and publication bias, and upgrades for large effect size, dose–response gradient, and attenuation by plausible confounding.

## Results

### Literature search

We totally identified 3903 articles in our initial search. We excluded 753 duplicates and additional 3125 articles by reviewing the title and abstract. A total of 25 full text were completely reviewed for eligibility. After full-text reviewing, we excluded six articles that were duplicate publications from the same studies^32–37^ and one study with insufficient data^38^. Finally, 18 prospective cohort studies were included^17–19,39–53^ (Supplementary Fig. 1).

### Characteristics of included studies

Characteristics of the included studies are provided in Supplementary Table 2. In total, 607,882 participants with an age range between 19 and 79 years were included. The length of the follow-up periods ranged from 3 to 24 years. Six studies were conducted in women^41,44,45,49–51^, three in men^19,39,47^, and the rest were in mixed. Six studies were conducted in the United States^39,41,44,45,50,51^, and 12 in other countries including, UK^40^, Australia^42,43^, Korea^17,18^, Japan^19,52^, Germany^46^, Finland^47^, China^49^ and Netherlands^48^. To assess dietary carbohydrate intake and LCDS, all studies used a food frequency questionnaire, except two studies that used a diet history questionnaire^19^ and a 7-day food diary^40^. Most studies controlled for important conventional confounders including physical activity (n = 18), smoking status (n = 17), energy intake (n = 17), BMI (n = 16), and alcohol consumption (n = 15). Only a few studies included in this meta-analysis did not adjust for energy intake^42^ and BMI^42,53^. Ten studies did not adjust for fiber intake^17,39,40,43,44,47,49,50,52,53^. Looking at the variation of NOS score, 13 studies out of 18 studies were rated high quality (NOS score of ≥ 7)^17,18,39–41,44–51^, and the others were rated to have moderate quality (NOS score of ≤ 7)^19,42,43,52,53^ (Supplementary Table 3) with none rated as low quality. Characteristics of primary cohort studies are presented in Supplementary Table 2 and reported effect sizes of type 2 diabetes across categories of dietary carbohydrate intake and LCDS are indicated in Supplementary Tables 4 and 5, respectively.

### Dietary carbohydrate and type 2 diabetes

**Figure 1:**
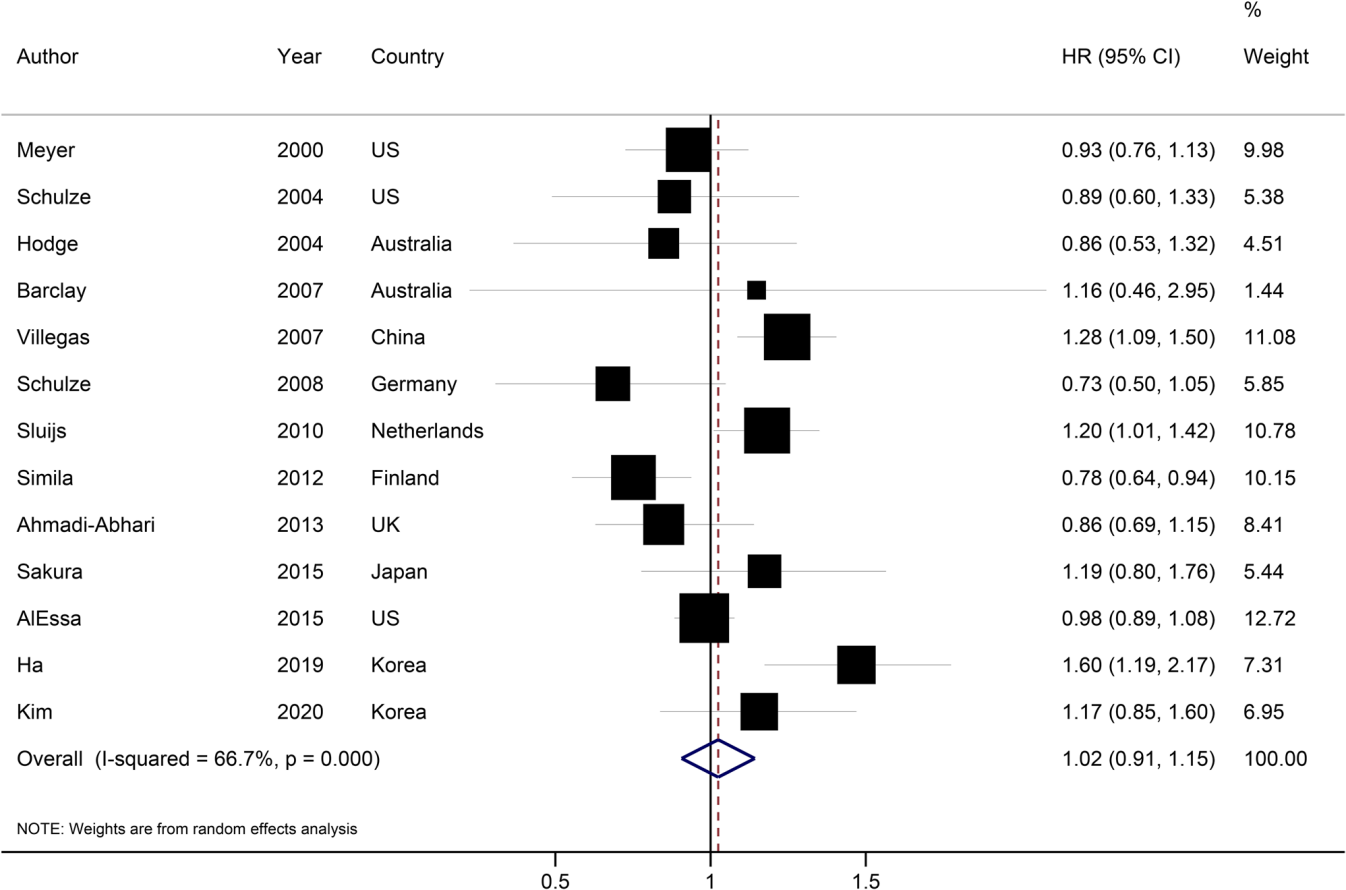
Hazard ratio of type 2 diabetes for the highest compared with the lowest category of type 2 diabetes. *HR* Hazard ratio.

Thirteen prospective cohort studies investigated the association between intake of carbohydrates from diet and T2D^17–19,40–49^. These studies included 403,883 participants, among whom 19,833 cases of T2D were found. In the main analysis, the highest compared with the lowest category of dietary carbohydrate intake was not associated with the risk of T2D (HR: 1.02, 95%CI: 0.91, 1.15; Fig. 1), with substantial heterogeneity (I^2^ = 67%, P_het_ < 0.001).

The association did not reach statistical significance by the stepwise exclusion of each primary study at a time (HR range: 0.99 to 1.05). In the subgroup analyses, there was no significant association across subgroups except for studies conducted in Asia (HR: 1.26, 95%CI: 1.11, 1.44; I^2^ = 6%, n = 6; Table 1). Geographical location, number of cases, and adjustment for dietary fiber intake were potential sources of heterogeneity. There was no evidence of small-study effect such as publication bias with Egger’s test (P = 0.99) and Begg’s test (P = 0.95) (Supplementary Fig. 2).

**Table 1:** Subgroup analyses of dietary carbohydrate and the risk of type 2 diabetes (highest versus lowest category meta-analysis).

	n	HR (95%CI)	I^2^, P_heterogeneity_	Chi-squared	P subgroup difference
All studies	13	1.02 (0.91, 1.15)	67%, < 0.001	36.05	–
**Sex**					0.82
Men	4	1.03 (0.76, 1.39)	72%, 0.01	10.79	
Women	6	1.06 (0.90, 1.24)	66%, 0.01	14.86	
Both	5	1.02 (0.83, 1.25)	52%, 0.08	8.34	
**Geographical region**					0.02
US + Europe	7	0.93 (0.82, 1.05)	58%, 0.27	14.28	
Asia	6	1.26 (1.11, 1.44)	6%, 0.38	5.48	
**Number of cases**					0.64
< 1000	8	1.00 (0.86, 1.16)	34%, 0.16	10.62	
> 1000	5	1.06 (0.87, 1.29)	84%, < 0.001	25.37	
**Follow-up duration**					0.67
< 10 years	6	0.99 (0.82, 1.20)	61%, 0.03	12.84	
> 10 years	7	1.05 (0.89, 1.24)	73%, 0.001	22.53	
**Adjustments**					
Smoking status					0.56
Yes	10	1.03 (0.91, 1.17)	69%, < 0.001	35.48	
No	1	0.84 (0.51, 1.15)	–	0.00	
Energy intake					0.81
Yes	10	1.02 (0.91, 1.15)	70%, < 0.001	35.98	
No	1	1.14 (0.43, 3.01)	–	0.00	
Body mass index					0.81
Yes	10	1.02 (0.91, 1.15)	70%, < 0.001	35.98	
No	1	1.14 (0.43, 3.01)	–	0.00	
Alcohol drinking					0.20
Yes	9	1.06 (0.94, 1.19)	64%, 0.002	27.48	
No	2	0.79 (0.66, 0.96)	0%, 0.45	0.67	
Fiber intake					0.94
Yes	7	1.04 (0.92, 1.17)	34%, 0.17	9.10	
No	6	1.02 (0.81, 1.27)	82%, < 0.001	26.94	

**Figure 2:**
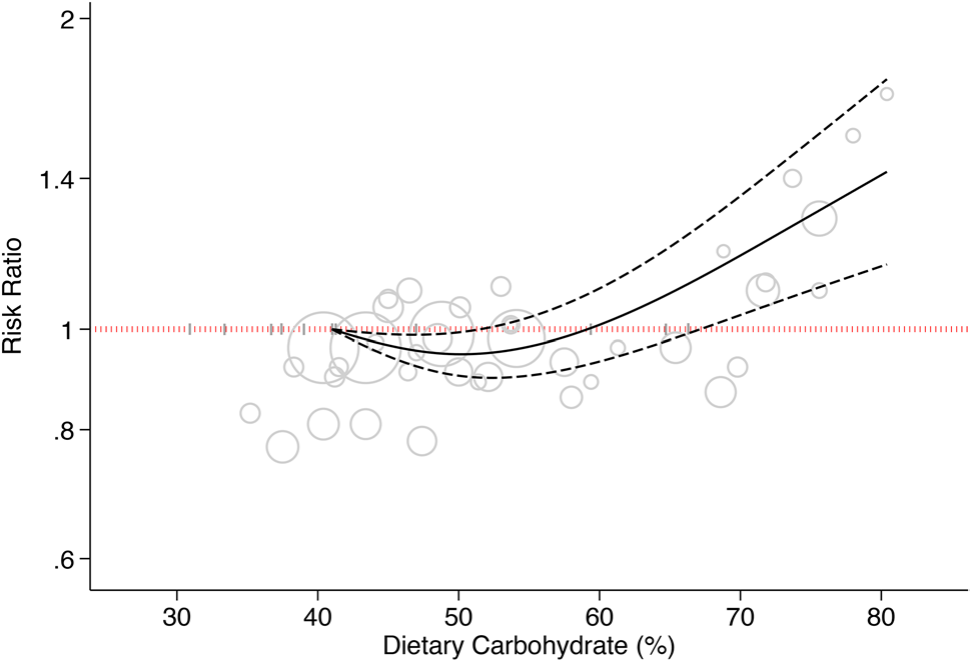
Dose–response association between carbohydrate intake and risk of type 2 diabetes. Solid line represents non-linear dose response and dotted lines represent 95% confidence interval. Circles represent hazard ratio point estimates for carbohydrate intake categories from each study with circle size proportional to inverse of standard error. Small vertical grey lines are baseline carbohydrate intake categories in each study.

All studies but one^18^ reported sufficient data for dose–response analysis. A 10% increment in energy intake form carbohydrate was not associated with the risk of T2D (HR: 1.02, 95%CI: 0.95, 1.09; I^2^ = 70%, Supplementary Fig. 3). Dose–response analysis indicated a J-shaped association between percentage energy from carbohydrate and the risk of T2D (P_nonlinearity_ < 0.001, P_dose-response_ < 0.001; Fig. 2), with the lowest risk at 50% energy from carbohydrate (HR_50%_: 0.95, 95%CI: 0.90, 0.99) and higher risk as carbohydrate intake increased. The HRs for 60%, 70%, and 80% calorie intake from carbohydrate were, respectively, 1.01 (95%CI: 0.93, 1.09), 1.18 (95%CI: 1.03, 1.35), and 1.41 (95%CI: 1.15, 1.73).

**Figure 3:**
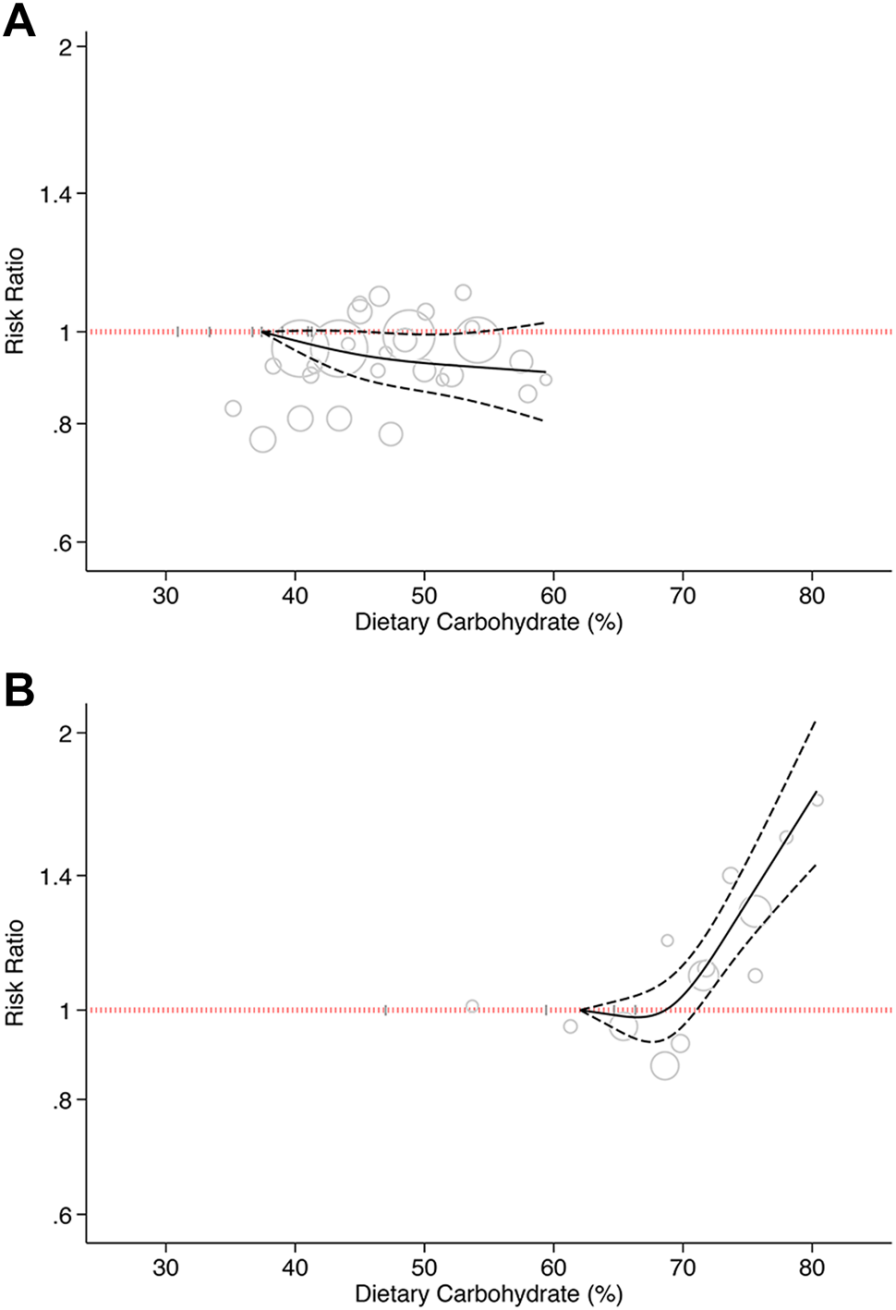
Dose–response association between carbohydrate intake and risk of type 2 diabetes. (**A**) Western countries. (**B**) Asian countries. Solid line represents non-linear dose response and dotted lines represent 95% confidence interval. Circles represent hazard ratio point estimates for carbohydrate intake categories from each study with circle size proportional to inverse of standard error. Small vertical grey lines are baseline carbohydrate intake categories in each study.

Restricting dose–response analyses to studies from Western countries only indicated that the risk of T2D did not change remarkably with increasing carbohydrate intake from 37 to 60% of total calorie (P_nonlinearity_ = 0.43, P_dose-response_ = 0.01.; n = 7, Fig. 3A). The HRs for 40%, 50%, and 60% calorie intake from carbohydrate in Western countries were 0.98 (95%CI: 0.95, 1.00), 0.93 (95%CI: 0.86, 0.99), and 0.90 (95%CI: 0.80, 1.02), respectively.

The analysis of Asian studies indicated that the risk of T2D did not change remarkably with increasing carbohydrate intake from 63 to 70% of total calorie (HR_70%_: 1.04, 95%CI: 0.96, 1.12), followed by a sharp and linear increment in risk (P_nonlinearity_ < 0.001, P_dose-response_ < 0.001; n = 5, Fig. 3B). The HR of T2D for carbohydrate intake of 80% total calorie was 1.70 (95%CI: 1.42, 2.02).

### Low-carbohydrate diet score and type 2 diabetes

Five studies investigated the association between LCDS and T2D^39,50–53^. These studies included 198,172 participants, among whom 9395 cases of T2D were found. There was no association between LCDS and the risk of T2D, either in the highest versus lowest category meta-analysis (HR: 1.14, 95%CI: 0.89, 1.47; I^2^ = 86%, n = 5; Supplementary Fig. 4), or in dose–response meta-analysis (HR_per 10-unit increase_: 1.06, 0.95%CI: 0.92, 1.21; I^2^ = 90%, n = 5; Supplementary Fig. 5). A non-significant association persisted in the subgroups defined by geographical location and follow-up duration (Supplementary Table 6).

### Grading the evidence

The certainty in the estimates was rated by the GRADE approach. The certainty of the evidence was rated low for dietary carbohydrate, with a downgrade for imprecision and an upgrade for dose–response gradient (Supplementary Table 7). The certainty in the estimates was rated very low for LCDS, with downgrades for imprecision and inconsistency.

## Discussion

This is the most recent and up-to-date meta-analysis of prospective cohort studies that examined the association between carbohydrate intake from diet and risk of T2D. Since the release of the three earlier meta-analyses^11–13^, some prospective cohort studies, especially those conducted in Asian countries, have been published that highlighted a need to present updated evidence for this association. We found evidence of a J-shaped relationship between carbohydrate intake and T2D in the non-linear dose–response, with the lowest risk at carbohydrate intake of 50% total calorie and with risk increasing significantly at 70% of total calorie. There appeared to be a marked difference in the association between carbohydrate intake and T2D between Asian and Western countries. Low carbohydrate diet score was not associated with the risk of T2D.

In line with ours, a previous meta-analysis on eight prospective studies in 2013 revealed that total carbohydrate intake was not associated with the risk of T2D in the linear dose–response analysis^12^. In addition, some earlier studies, mostly conducted in Western countries, did not find an association between carbohydrate intake from diet and the risk of T2D^32,42,44,45^.

Another recent meta-analysis of cohort studies showed a non-significant association between carbohydrate intake and the risk of T2D in Western countries and in contrast, found a significant positive association in one Asian study^11^. We updated the evidence and included additional recent studies conducted in Asian countries which showed that carbohydrate intake, within the recommended daily intake of 45–65% of total calorie, as reported in Western countries, was not associated with an increased risk of type 2 diabetes, and even was associated with a modest lower risk at 50% carbohydrate intake. However, the nonlinear dose–response meta-analysis of five Asian studies suggested that carbohydrate intake higher than 70% of total calorie was strongly associated with a higher risk of T2D.

A recent meta-analysis of prospective cohort studies found a similar U-shaped association between carbohydrate intake and total mortality, with the lowest risk being found at 50–55% of carbohydrate intake, and an increased risk at an intake of more than 70% carbohydrate intake^54^. Evidence from earlier prospective cohort studies evaluating the association between the quality and quantity of dietary carbohydrates, reflected by dietary glycemic index and load, suggests that both quality and quantity of dietary carbohydrates are associated with the risk of T2D^18,43,55,56^. In addition, there was also evidence of a causal association between dietary glycemic index and load and the risk of T2D^56,57^.

Studies have suggested some mechanisms relating dietary carbohydrates to the risk of T2D. The long-term exposure to dietary carbohydrates may provide a continuous signal to the pancreatic β-cell to secret insulin to reduce blood glucose levels. Consequently, β-cell exhaustion can result in glucose intolerance^58^. Furthermore, excessive carbohydrates intake produces a large amount of acetyl CoA in the metabolic pathways, thus releasing lots of free radical and thereby exacerbating insulin resistance^58,59^.

There are also several explanations for the observed geographical difference found in the present study. First and most importantly, carbohydrate intake is substantially higher in Asian countries (generally > 60%) than in Western countries (generally < 50%)^54^. We found a relatively J-shaped association, wherein the US and European countries mainly represented the left side of the curve and in contrast, Asian countries represented the right side of the curve^54^. Higher carbohydrate intake increases demand for insulin secretion, leading to β-cell exhaustion. Second, Asian populations have a lower capacity of insulin secretion than that of their Western counterparts^60–62^. In addition, type of carbohydrate consumed, especially proportion of whole and refined grains, may be different across the globe and this may create a difference in the association between dietary carbohydrates with the risk of T2D. The main source of carbohydrates in most Asian countries is refined carbohydrates such as white rice and bread, reflecting low diet quality^63,64^. White rice, a high glycemic index food, was associated with an increased risk of T2D, especially in Asian societies^65,66^. More recently, a prospective cohort study conducted in 21 countries across the globe indicated that higher rice consumption was associated with an increased risk of type 2 diabetes in South Asian countries, and a modest non-significant association in other regions^16^.

### Strength and limitations

We updated previous meta-analyses and included the most recent studies, especially those conducted in Asia. We included new Asian articles and looked at them separately by subgrouping them because of the difference in their diet. Here we showed that higher carbohydrate intake more than the recommended daily intake of 45–65% was strongly associated with the risk of T2D. We applied a newly-developed one-stage linear mixed effects meta-analysis that creates more efficient and flexible plots than the conventional two-stage model.

Some limitations should be noted in the context of our findings. Due to the observational nature of the studies included, our resulting associations cannot establish causality. According to the GRADE, the certainty of the evidence was rated low for dietary carbohydrate and very low for LCDS. In addition, we had insufficient data for the analysis of LCDS. We used total carbohydrate intake as exposure which represents a large diverse group of foods such as whole and refined grains. The potential difference in foods constituting total carbohydrate intake in Asian and Western countries might confound the association between total carbohydrate intake and T2D.

## Conclusion

The results of this updated meta-analysis of 18 cohort studies (607,882 participants with 29,228 cases) showed that carbohydrate intake within the recommended dietary intake of 45% to 65% of total calorie was not associated with a higher risk of T2D and even was associated with a modest lower risk at 50% carbohydrate intake. Carbohydrate intake more than 70% of total calorie, as found in Asian countries, was associated with substantial higher risk of T2D. However, these findings were obtained from observational studies and thus, could not prove causality. More research, especially in Asian countries, is needed to investigate the association between carbohydrate intakes higher than recommended dietary intake with the risk of T2D.

## Supplementary Information

Supplementary Information.

## Data Availability

The data, codes, analytical syntax, and other additional data used for the present meta-analysis are available from the corresponding author on reasonable request.

## Abbreviations

GRADE: Grading of recommendations assessment, development, and evaluation (GRADE) approach
LCDS: Low carbohydrate diet score
