# Supplementary material for: Dietary carbohydrate and the risk of type 2 diabetes: an updated systematic review and dose–response meta-analysis of prospective cohort studies
Source: Sci Rep. 2022 Feb 15;12:2491. doi: 10.1038/s41598-022-06212-9 (PMC8847553; doi:10.1038/s41598-022-06212-9)
Supplement: Supplementary file 1 — Supplementary Information. [file 41598_2022_6212_MOESM1_ESM.docx]

**Dietary carbohydrate and the risk of type 2 diabetes: an updated systematic review and dose-response meta-analysis of prospective cohort studies**

Supplementary Information including Supplementary Tables 1-7, Supplementary Figures 1-5, and MOOSE checklist

**Supplementary** **Table 1**: Medical subject headings (MeSH) and non-MeSH keywords used to search for potential relevant publications.

| **Concept 1 (exposure)** | Carbohydrates[Mesh] OR Carbohydrates[tiab] "Dietary Carbohydrates" [Mesh] OR "Dietary Carbohydrates"[tiab] OR "Diet, Carbohydrate-Restricted"[Mesh] OR "Carbohydrate-Restricted Diet"[tiab] OR "Low-Carbohydrate Diets"[tiab] OR "Diet, High-Protein Low-Carbohydrate"[Mesh] OR "High Protein Low Carbohydrate Diet"[tiab] OR "Atkins Diet"[tiab] OR "High-Protein Carbohydrate-Restricted Diet"[tiab] OR "South Beach Diet"[tiab] OR Diet, Ketogenic[Mesh] OR "Ketogenic Diet"[tiab] OR "Modified Atkins diet"[tiab] OR Ketosis[Mesh] OR Ketosis[tiab] |
| --- | --- |
| **Concept 2 (outcome)** | "Diabetes Mellitus"[Mesh] OR "Diabetes Mellitus"[tiab] OR "Diabetic patients"[tiab] OR DM[tiab] OR "Diabetes Mellitus, Type 2"[Mesh] OR "Diabetes Mellitus, Type 2"[tiab] OR "Diabetes Mellitus, Noninsulin-Dependent"[tiab] OR "Diabetes Mellitus, Ketosis-Resistant"[tiab] OR "Non-Insulin-Dependent Diabetes Mellitus"[tiab] OR "NIDDM"[tiab] OR "Diabetes Mellitus"[tiab] OR "MODY"[tiab] OR "T2DM"[tiab] |
| **Concept 3 (study design)** | "population-based"[tiab] OR prospective[tiab] OR "case control"[tiab] OR longitudinal[tiab] OR follow-up[tiab] OR cohort[tiab] OR retrospective[tiab] OR "Longitudinal Studies"[Mesh] OR "Prospective Studies"[Mesh] OR "Case-Control Studies"[Mesh] OR "Cohort Studies"[Mesh] OR "Retrospective Studies"[Mesh] |

**Supplementary** **Table 2**. Characteristics of prospective cohort studies included in the meta-analysis of dietary carbohydrat and the risk of type 2 diabetes.

| Author, year | Study | Country | Sample size | Female, % | Age range, mean (years) | Follow-up | Exposure (identification method) | Adjustments |
| --- | --- | --- | --- | --- | --- | --- | --- | --- |
| Ahmadi-Abhari, 2013 | EPIC-Norfolk | UK | 3496 | 80% | 40-79 | 10 years | CHO (7-day food diary) | Age, sex, total energy intake, BMI, family history of type 2 diabetes, cigarette smoking, alcohol intake, physical activity and level of education |
| AlEssa, 2015 | the Nurses’ Health Study | US | 70,025 | 100% | 30-55 | 24 years | CHO (FFQ with 133 items) | Age, BMI, family history of diabetes, postmenopausal status, smoking status, alcohol intake, physical activity level, multivitamin use, race, and total energy intake, red meat, coffee, magnesium, ratio of polyunsaturated fat to saturated fat, and trans-fat, cereal fiber, sugar-sweetened beverages, and fruit and vegetables |
| Barclay, 2007 | Blue Mountains Study | Australia | 1833 | 56% | ≥49 (65) | 10 years | CHO (FFQ with 145 items) | Age, sex, family history of diabetes, smoking, triglycerides, HDL cholesterol, and physical activity |
| Bao,  2016 | the Nurses’ Health Study II | US | 4502 | 100% | 24-44 | 12 years | LCDS (FFQ) | Age, parity, age at first birth, race/ethnicity, family history of diabetes, oral contraceptive use, menopausal status, cigarette smoking, alcohol intake, physical activity, total energy intake, and glycemic index |
| de Koning, 2011 | Health Professionals Follow-Up study | US | 40,475 | 0% | 40-75 | 20 years | LCDS (FFQ with 131 items) | Age, smoking, physical activity, coffee intake, alcohol intake, family history of T2D, total energy intake, and BMI |
| Ha,  2019 | Korean Genome and Epidemiology Study | Korea | 11,190 | 50% | 40-69 | 11.5 years | CHO (FFQ with 103 items) | Alcohol consumption, BMI, education level, household income level, marital status, smoking status, parental history of diabetes, physical activity, residence, protein intake, total energy intake, and fasting blood glucose at baseline |
| Halton, 2008 | the Nurses’ Health Study | US | 85,059 | 100% | 30–55 | 20 years | LCDS (FFQ with 61 items) | Age, smoking, postmenopausal hormone use, physical activity, alcohol intake, family history of type 2 diabetes in a first-degree relative, and BMI |
| Hodge, 2004 | the Melbourne Collaborative Cohort Study | Australia | 31,641 | 59% | 40-69 (54) | 4 years | CHO (FFQ with 121 items) | Age, sex, country of birth, physical activity, family history of diabetes, alcohol intake, education level, weight change in the last 5 years, and energy intake |
| Kim,  2020 | the MultiRural Communities Cohort | Korea | 8310 | 63.5% | ≥40 | 6 years | CHO (FFQ with 106 items) | Age, educational level, smoking status, regular exercise, alcohol consumption, BMI, and intakes of cereal fiber, red meat, milk, coffee, and folate |
| Meyer, 2000 | the Iowa Women’s Health Study | US | 35,988 | 100% | 55-69 (61) | 6 years | CHO (FFQ with 127 items) | Age, total energy intake, BMI, waist-to-hip ratio, education, pack-years of smoking, alcohol intake, and physical activity |
| Nanri, 2015 | Japan Public Health Center-Based Prospective Study | Japan | 64,674 | 57.01% | 45-75 | 5 years | LCDS (FFQ with 147 items) | Age, study area, smoking status, alcohol consumption, family history of diabetes mellitus, total physical activity, history of hypertension, total energy intake, and coffee consumption |
| Sakurai, 2015 | Toyama Prefecture Cohort Study | Japan | 2006 | 0% | 35-55 | 10 years | CHO (DHQ) | Age, a family history of diabetes, smoking, alcohol consumption, regular exercise and performance of shift work, total energy intake and total fiber intake, and the presence of hypertension and hyperlipidemia at baseline |
| Sali,  2020 | Tehran Lipid and Glucose Study | Iran | 4351 | 55.6% | >19 (40.5) | 3 years | LCDS (FFQ with 168 items) | Age, sex, waist circumference, physical activity, educational level, smoking, daily energy intake, and family history of diabetes |
| Schulze, 2004 | the Nurses’ Health Study II | US | 91,249 | 100% | 24-44 | 8 years | CHO (FFQ with 133 items) | Age, BMI, energy intake, alcohol intake, physical activity, family history of diabetes, smoking, history of high blood pressure, history of high blood cholesterol, postmenopausal hormone use, oral contraceptive use, intakes of cereal fiber, magnesium, and caffeine |
| Schulze, 2008 | EPIC-Potsdam | Germany | 25,067 | 38% | 35–65 | 7 years | CHO (FFQ with 148 items) | Age, education, occupational activity, sport activity, cycling, smoking, alcohol intake, and total energy intake, fiber intake, Mg intake, PUFA:SFA ratio, and MUFA:SFA ratio |
| Simila, 2012 | the Alpha-Tocopherol, Beta-Carotene Cancer Prevention Study | Finland | 25,943 | 0% | 50-69 | 12 years | CHO (FFQ with 276 items) | Age and intervention group, BMI, energy and coffee consumption |
| Sluijs, 2010 | EPIC-NL | Netherlands | 37,846 | 74% | 21–70 (51) | 10 years | CHO (FFQ with 79 items) | Age, Sex, energy-adjusted alcohol consumption, physical activity, waist circumference, BMI, smoking status, mean systolic blood pressure, educational level, family history of diabetes |
| Villegas, 2007 | the Shanghai Women’s Health Study | China | 64,227 | 100% | 40-70 | 4.6 years | CHO (FFQ with 77 items) | Age, BMI, smoking, activity, alcohol, and energy intake |
| Abbreviations: CHO, carbohydrate; DHQ, diet history questionnaire; EPIC, European Prospective Investigation into Cancer and Nutrition; FFQ, food frequency questionnaire; HDL, high density lipoprotein cholesterol; LCDS, low carbohydrate diet score; MUFA, monounsaturated fats; PUFA, polyunsaturated fats; SFA, saturated fats; T2D, type 2 diabetes. | | | | | | | | |

**Supplementary** **Table 3**: Quality assessment of prospective cohort studies included in the systematic review and meta-analysis based on Newcastle-Ottawa scale (NOS).

| study | Representativeness of the exposed cohort | Selection of the non-exposed cohort | Ascertainment of exposure | Outcome of interest was not present at start of study | Age and sex adjustments | Controls for any additional factors* | Assessment of outcome | Follow-up long enough** | Adequacy of follow-up of cohorts | Total |
| --- | --- | --- | --- | --- | --- | --- | --- | --- | --- | --- |
| Ahmadi-Abhari, 2013 | * | * |  | * | * | * | * | * | * | 8 |
| AlEssa, 2015 | * | * | * | * | * | * | * | * | * | 9 |
| Barclay, 2007 |  | * | * | * | * |  |  | * | * | 6 |
| Ha, 2019 | * | * | * | * |  | * | * | * | * | 8 |
| Hodge, 2004 | * | * | * | * |  |  | * |  | * | 6 |
| Kim, 2020 | * | * | * | * |  |  | * | * | * | 7 |
| Meyer, 2000 | * | * | * | * | * |  |  | * | * | 7 |
| Sakurai, 2015 |  | * |  | * | * |  | * | * | * | 6 |
| Schulze, 2004 | * | * | * | * | * | * | * | * | * | 9 |
| Schulze, 2008 | * | * | * | * |  |  | * | * | * | 7 |
| Simila, 2012 |  | * | * | * | * |  | * | * | * | 7 |
| Sluijs, 2010 | * | * | * | * | * | * | * | * | * | 9 |
| Villegas, 2007 | * | * | * | * | * | * |  |  | * | 7 |
| Nanri, 2015 | * | * | * | * |  |  | * | * |  | 6 |
| Halton, 2008 | * | * | * | * | * |  | * | * | * | 8 |
| Bao, 2016 | * | * | * | * | * |  | * | * | * | 8 |
| Sali, 2020 |  | * | * | * | * |  | * |  |  | 5 |
| de Koning, 2011 | * | * | * | * | * |  | * | * | * | 8 |
| * Adjustment for body mass index, alcohol drinking, physical activity, family history of diabetes, and fiber and energy intakes.  ** At least five years. | | | | | | | | | | |

**Supplementary** **Table 4.** Reported effect size of type 2 diabetes across categories of dietary carbohydrate intake in prospective cohort studies.

| **Dietary carbohydrate** | **Effect size (95%CI)** |
| --- | --- |
| **Ahmadi Abhari, 2014** | |
| 41.0 (median, % calorie) | 1.0 |
| 46.5 | 1.07 (0.78, 1.42) |
| 50.0 | 0.91 (0.75, 1.12) |
| 53.4 | 1.10 (0.80, 1.43) |
| 58.1 | 0.86 (0.69, 1.15) |
| Per 70 g/d | 0.82 (0.66, 1.03) |
| **AlEssa, 2015** | |
| 159.0 (median, g/d) | 1.0 |
| 181.0 | 0.96 (0.89, 1.04) |
| 195.0 | 0.96 (0.89, 1.04) |
| 208.3 | 0.99 (0.91, 1.08) |
| 228.4 | 0.98 (0.89, 1.08) |
| **Ha, 2019 (Male)** | |
| 64.7 (median, % calorie) | 1.0 |
| 69.8 | 0.92 (0.69, 1.22) |
| 73.7 | 1.40 (1.02, 1.93) |
| 78 | 1.54 (1.03, 2.30) |
| **Ha, 2019 (Female)** | |
| 66.3 (median, % calorie) | 1.0 |
| 71.8 | 1.11 (0.82,1.51) |
| 75.6 | 1.09 (0.75, 1.57) |
| 80.4 | 1.69 (1.08, 2.67) |
| **Meyer, 2000** | |
| 176 (median, g/d) | 1.0 |
| 202 | 1.05 (0.87, 1.26) |
| 218 | 0.98 (0.81, 1.19) |
| 234 | 0.90 (0.74, 1.09) |
| 259 | 0.93 (0.76, 1.13) |
| **Sakura, 2015** | |
| <50 (range, % calorie) | 1.0 |
| 50-57.4 | 1.0 (0.65, 1.53) |
| 57.5-65 | 0.96 (0.68, 1.47) |
| >65.0 | 1.19 (0.77, 1.87) |
| **Schulze, 2004** | |
| 41.3 (median, % calorie) | 1.0 |
| 46.5 | 1.09 (0.87, 1.37) |
| 50.1 | 1.05 (0.80, 1.38) |
| 53.7 | 1.01 (0.74, 1.39) |
| 59.4 | 0.89 (0.60, 1.33) |
| **Schulze, 2008 (Male)** | |
| 30.9 (median, % calorie) | 1.0 |
| 35.2 | 0.83 (0.62, 1.10) |
| 38.3 | 0.92 (0.69, 1.23) |
| 41.5 | 0.92 (0.68, 1.25) |
| 46.4 | 0.91 (0.66, 1.26) |
| **Schulze, 2008 (Female)** | |
| 36.7 (median, % calorie) | 1.0 |
| 41.2 | 0.90 (0.64, 1.13) |
| 44.1 | 0.97 (0.69, 1.36) |
| 47 | 0.95 (0.66, 1.35) |
| 51.4 | 0.89 (0.62, 1.29) |
| **Simila, 2012** | |
| 33.4 (median, % calorie) | 1.0 |
| 37.5 | 0.77 (0.65, 0.92) |
| 40.4 | 0.81 (0.68, 0.97) |
| 43.4 | 0.81 (0.68, 0.98) |
| 47.4 | 0.78 (0.64, 0.94) |
| **Villegas, 2007** | |
| 263.5 (median, g/d) | 1.0 |
| 269.1 | 0.96 (0.80, 1.15) |
| 276.3 | 0.87 (0.73, 1.05) |
| 287.1 | 1.09 (0.92, 1.29) |
| 337.6 | 1.28 (1.09, 1.50) |

**Supplementary** **Table 5.** Reported effect size of type 2 diabetes across categories of low carbohydrate diet score in prospective cohort studies.

| **Low carbohydrate diet score** | **Effect size (95%CI)** |
| --- | --- |
| **Bao, 2016** | |
| 5 (score) | 1.0 |
| 10 | 0.95 (0.70, 1.30) |
| 15 | 1.02 (0.77, 1.36) |
| 19 | 1.28 (0.96, 1.71) |
| 25 | 1.36 (1.04, 1.78) |
| **de Koning, 2011** | |
| 5 (score) | 1.0 |
| 11 | 1.03 (0.89, 1.19) |
| 15 | 1.14 (0.99, 1.31) |
| 19 | 1.17 (1.02, 1.33) |
| 25 | 1.31 (1.14, 1.49) |
| **Halton, 2008** | |
| 5 (score) | 1.0 |
| 10.5 | 0.98 (0.85,1.12) |
| 14 | 0.96 (0.84, 1.10) |
| 17 | 0.92 (0.81,1.05) |
| 26 | 0.90 (0.78, 1.04) |
| **Nanri, 2015 (Men)** | |
| 3 (score) | 1.0 |
| 9 | 1.09 (0.85, 1.40) |
| 13 | 1.16 (0.92, 1.48) |
| 18 | 1.10 (0.86, 1.42) |
| 24 | 1.0 (0.77, 1.30) |
| **Nanri, 2015 (Women)** | |
| 5 (score) | 1.0 |
| 12 | 0.93 (0.72, 1.20) |
| 17 | 0.71 (0.54, 0.94) |
| 21 | 0.71 (0.53, 0.97) |
| 26 | 0.63 (0.46, 0.84) |

**Supplementary** **Table 6.** Subgroup analyses of low carbohydrate diet score and the risk of type 2 diabetes (highest versus lowest category meta-analysis).

|  |  | n | HR (95%CI) | I^2^, P_heterogeneity_ | Chi-squared | P subgroup difference |
| --- | --- | --- | --- | --- | --- | --- |
| All studies |  | 5 | 1.14 (0.89, 1.47) | 86%, <0.001 | 29.00 | - |
| Sex |  |  |  |  |  | 0.27 |
| Men |  | 1 | 1.00 (0.77, 1.30) | - | 0.00 |  |
| Women |  | 4 | 1.01 (0.75, 1.37) | 90%, <0.001 | 29.28 |  |
| Both |  | 1 | 2.16 (1.16, 4.03) | - | 0.00 |  |
| Geographical region | | |  |  |  | 0.98 |
| US |  | 3 | 1.16 (0.88, 1.53) | 88%, <0.001 | 16.12 |  |
| Asia |  | 2 | 1.27 (0.49, 3.27) | 88%, 0.004 | 8.39 |  |
| Follow-up duration |  |  |  |  |  | 0.98 |
| <10 years |  | 3 | 1.16 (0.88, 1.53) | 88%, <0.001 | 8.39 |  |
| >10 years |  | 2 | 1.27 (0.49, 3.27) | 88%, 0.004 | 16.12 |  |

**Supplementary** **Table 7**. GRADE evidence table for association of dietary carbohydrate, low-carbohydrate diet score, and the risk of type 2 diabetes.

**Author(s)**: Fateme Hosseini, Ahmad Jayedi, Tauseef Ahmad Khan, Sakineh Shab-Bidar

**Question**: Is dietary carbohydrate intake associated with the risk of type 2 diabetes?

**Setting**: General population

**Bibliography**:

| **Certainty assessment** | | | | | | | **№ of patients** | | **Effect** | | **Certainty** | **Importance** |
| --- | --- | --- | --- | --- | --- | --- | --- | --- | --- | --- | --- | --- |
| **№ of studies** | **Study design** | **Risk of bias** | **Inconsistency** | **Indirectness** | **Imprecision** | **Other considerations** | **Participants** | **Cases**  **(event rate)** | **Relative (95% CI)** | **Absolute (95% CI)** |  |  |
| **Dietary carbohydrate (follow up: range 4 years to 24 years)** | | | | | | | | | | | | |
| 13 | observational studies | not serious | not serious ^a^ | not serious | serious ^b^ | dose response gradient | 403,883 | 19,833 (4.9%) | **HR 1.02** (0.91 to 1.15) | **1 more per 1,000** (from 4 fewer to 7 more) | ⨁⨁◯◯ LOW | CRITICAL |
| **Low-carbohydrate diet score (follow up: range 3 years to 20 years)** | | | | | | | | | | | | |
| 5 | observational studies | not serious | serious ^c^ | serious ^d^ | serious ^e^ | none | 198,172 | 9395 (4.7%) | **HR 1.14** (0.89 to 1.47) | **6 more per 1,000** (from 5 fewer to 21 more) | ⨁◯◯◯ VERY LOW | CRITICAL |

**CI:** Confidence interval; **HR:** Hazard Ratio

#### Explanations

a. I^2^=67%, Phet<0.001. The 95%CI of most studies overlapped, and the HR for the subgroup of Asian studies was 1.26 (95%CI: 1.12, 1.44; I^2^=6%, n=6). Not downgraded.

b. The 95%CI include the null value (HR: 1.00), and the upper bound of the 95%CI was >1.10. Downgraded.

c. I^2^=86%, Phet<0.001. Substantial heterogeneity that was not explained by the sensitivity and subgroup analyses. Downgraded.

d. Serious indirectness since three studies (>75% of participants) were conducted in health professionals. Downgraded.

e. The 95%CI include the null value (HR: 1.00) and the upper bound of the 95%CI was >1.10. Downgraded.

Records excluded (n =3125)

Studies included in quantitative synthesis (meta-analyses)
(n = 18)

Studies included in qualitative synthesis
(n = 18)

Full-text articles assessed for eligibility
(n = 25)

Records screened
(n = 3150)

Records after duplicates removed
(n = 3150)

Additional records identified through other sources
(n = 0)

## Identification

## Eligibility

## Included

## Screening

Records identified through database searching
(n =3903)

Full-text articles excluded, with reasons (n =25):

Duplicate studies (n=6)

No sufficient data (n=1)

**Supplementary** **Figure 1**. Literature search and study section process.

**Supplementary** **Figure 2.** Funnel plot of the relative risks of 13 studies on dietary carbohydrate and the risk of type 2 diabetes. Begg’s test P=0.95, Egger’s test P=0.99. Log RR: natural logarithm of relative risk. s.e: standard error.


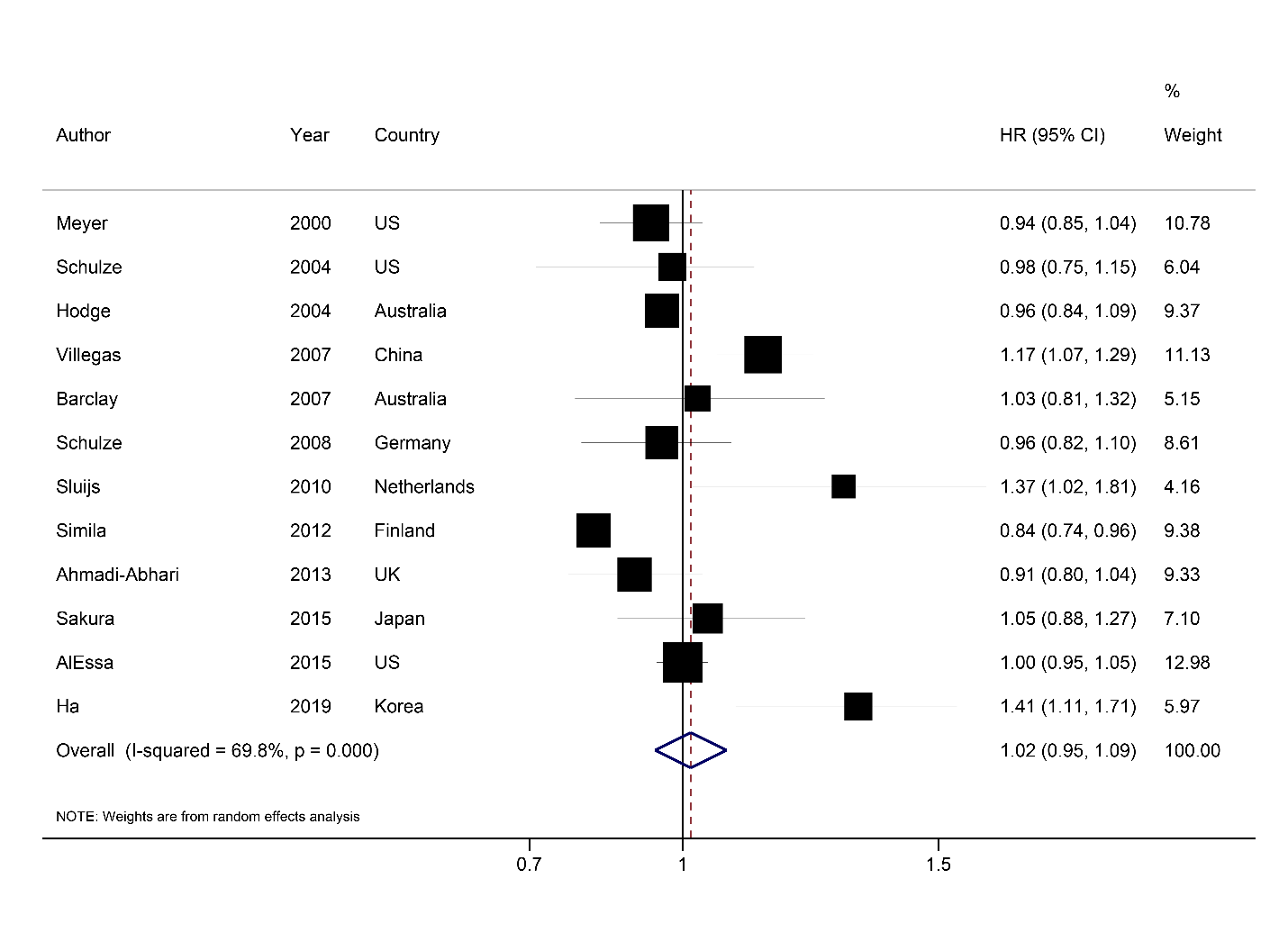


**Supplementary** **Figure 3**. Hazard ratio of type 2 diabetes for a 10% increment in energy intake from carbohydrate.


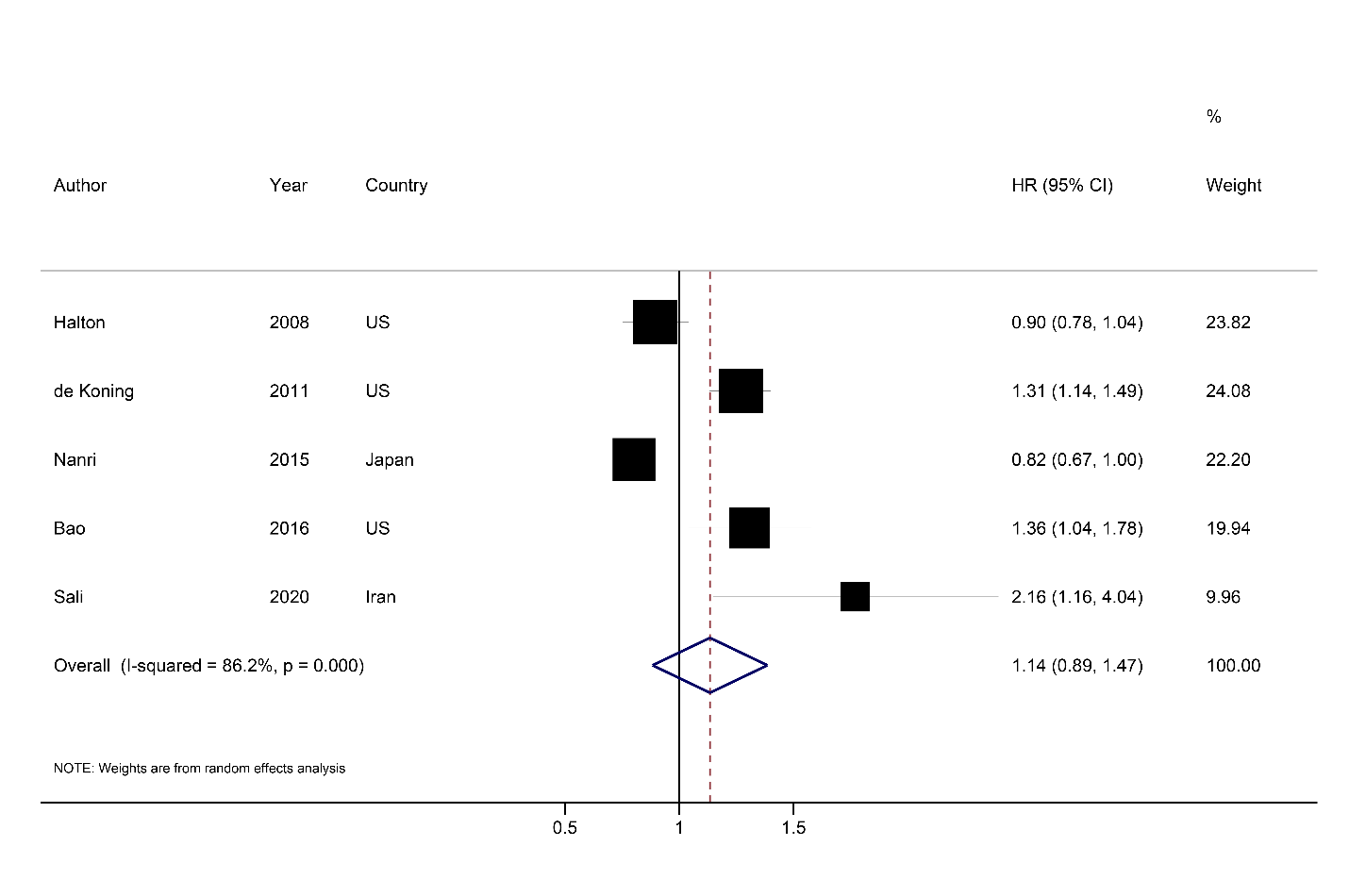


**Supplementary** **Figure 4**. Hazard ratio of type 2 diabetes for the highest compared with lowest category of low carbohydrate diet score.


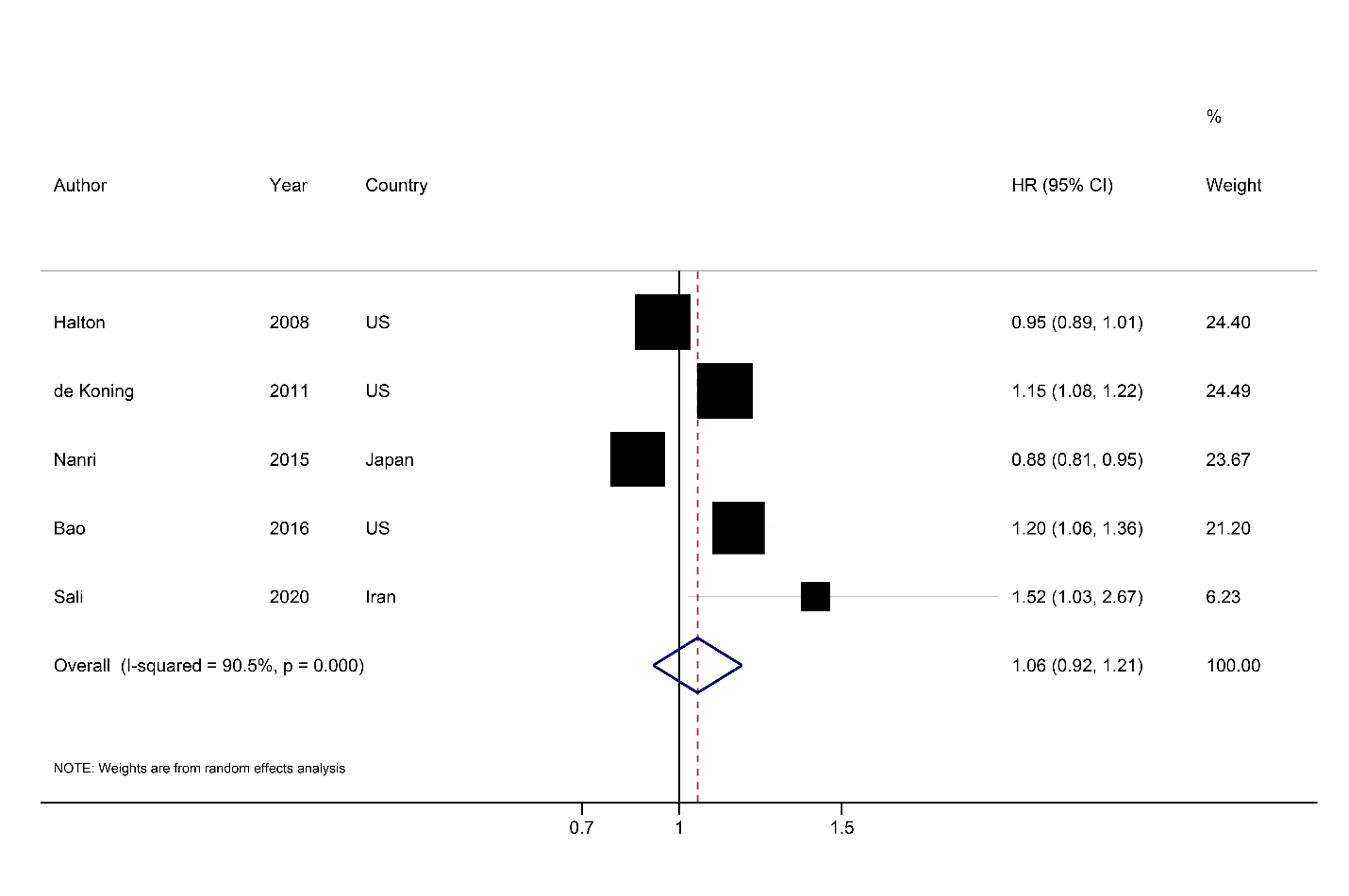


**Supplementary** **Figure 5**. Hazard ratio of type 2 diabetes for a 10-unit increase in low carbohydrate diet score.

**MOOSE Checklist for Meta-analyses of Observational Studies**

| **Item No** | **Recommendation** | **Reported on Page No** |
| --- | --- | --- |
| Reporting of background should include | | |
| 1 | Problem definition | 2 |
| 2 | Hypothesis statement | 3 |
| 3 | Description of study outcome(s) | 4 |
| 4 | Type of exposure or intervention used | 4 |
| 5 | Type of study designs used | 4 |
| 6 | Study population | 4 |
| Reporting of search strategy should include | | |
| 7 | Qualifications of searchers (eg, librarians and investigators) | Title page |
| 8 | Search strategy, including time period included in the synthesis and key words | 4 |
| 9 | Effort to include all available studies, including contact with authors | 4 |
| 10 | Databases and registries searched | 4 |
| 11 | Search software used, name and version, including special features used (eg, explosion) | NA |
| 12 | Use of hand searching (eg, reference lists of obtained articles) | 4 |
| 13 | List of citations located and those excluded, including justification | Supp Fig 1 |
| 14 | Method of addressing articles published in languages other than English | 4 |
| 15 | Method of handling abstracts and unpublished studies | NA |
| 16 | Description of any contact with authors | No need |
| Reporting of methods should include | | |
| 17 | Description of relevance or appropriateness of studies assembled for assessing the hypothesis to be tested | 5 |
| 18 | Rationale for the selection and coding of data (eg, sound clinical principles or convenience) | 5 |
| 19 | Documentation of how data were classified and coded (eg, multiple raters, blinding and interrater reliability) | 5 |
| 20 | Assessment of confounding (eg, comparability of cases and controls in studies where appropriate) | 6-7 |
| 21 | Assessment of study quality, including blinding of quality assessors, stratification or regression on possible predictors of study results | 6-7 |
| 22 | Assessment of heterogeneity | 6-7 |
| 23 | Description of statistical methods (eg, complete description of fixed or random effects models, justification of whether the chosen models account for predictors of study results, dose-response models, or cumulative meta-analysis) in sufficient detail to be replicated | 6-7 |
| 24 | Provision of appropriate tables and graphics | Table 1, Figures 1-3, and Supplements |
| Reporting of results should include | | |
| 25 | Graphic summarizing individual study estimates and overall estimate | Figure S1 |
| 26 | Table giving descriptive information for each study included | Table S2 |
| 27 | Results of sensitivity testing (eg, subgroup analysis) | Table S5, Pages 8-0 |
| 28 | Indication of statistical uncertainty of findings | 8-10 |

| **Item No** | **Recommendation** | **Reported on Page No** |
| --- | --- | --- |
| Reporting of discussion should include | | |
| 29 | Quantitative assessment of bias (eg, publication bias) | 10 |
| 30 | Justification for exclusion (eg, exclusion of non-English language citations) | - |
| 31 | Assessment of quality of included studies | Table S2 |
| Reporting of conclusions should include | | |
| 32 | Consideration of alternative explanations for observed results | Pages 10-13 |
| 33 | Generalization of the conclusions (ie, appropriate for the data presented and within the domain of the literature review) | Pages 10-13 |
| 34 | Guidelines for future research | 13 |
| 35 | Disclosure of funding source | 14 |
